# Foraging Fidelity as a Recipe for a Long Life: Foraging Strategy and Longevity in Male Southern Elephant Seals

**DOI:** 10.1371/journal.pone.0032026

**Published:** 2012-04-10

**Authors:** Matthieu Authier, Ilham Bentaleb, Aurore Ponchon, Céline Martin, Christophe Guinet

**Affiliations:** 1 Centre d'Études Biologiques de Chizé, UPR 1934 du CNRS, Villiers-en-Bois, France; 2 École Doctorale Sciences pour l'Environnement Gay Lussac-Université de Poitiers, France; 3 Institut des Sciences de l'Evolution, UMR 5554 du CNRS, Montpellier, France; 4 Centre d'Écologie Fonctionnelle et Évolutive, UMR 5175 du CNRS, Montpellier, France; Phillip Island Nature Parks, Australia

## Abstract

Identifying individual factors affecting life-span has long been of interest for biologists and demographers: how do some individuals manage to dodge the forces of mortality when the vast majority does not? Answering this question is not straightforward, partly because of the arduous task of accurately estimating longevity in wild animals, and of the statistical difficulties in correlating time-varying ecological covariables with a single number (time-to-event). Here we investigated the relationship between foraging strategy and life-span in an elusive and large marine predator: the Southern Elephant Seal (*Mirounga leonina*). Using teeth recovered from dead males on îles Kerguelen, Southern Ocean, we first aged specimens. Then we used stable isotopic measurements of carbon (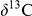) in dentin to study the effect of foraging location on individual life-span. Using a joint change-point/survival modelling approach which enabled us to describe the ontogenetic trajectory of foraging, we unveiled how a stable foraging strategy developed early in life positively covaried with longevity in male Southern Elephant Seals. Coupled with an appropriate statistical analysis, stable isotopes have the potential to tackle ecological questions of long standing interest but whose answer has been hampered by logistic constraints.

## Introduction

Identifying individual factors affecting life-span has long been of interest for biologists and demographers [1], [2] : how do some individuals manage to dodge the “little devils" of death [3] longer than the large majority of their conspecifics? To start answering this question, several problems need to be overcome; the first being the accurate estimation of life-span or longevity. In the case of vertebrates, longevity may be estimated by following individuals from their birth till their death using mark-recapture methods [4]. However, long-lived organisms (for example, seabirds) present additional challenges: a wandering albatross (*Diomedea exulans*) may live up to 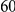 years [5], requiring a lot of patience, serendipity, and skills to secure funds and manpower from demographers. Yet knowledge of individual longevity may be critical to shed light on life-history patterns [6]–[12]. Recent studies have moved away from population-level (life-tables, for example [13]) to individual-level inferences (for example, [14]), which is the level where natural selection occurs. This move is the result of both conceptual and technical advances, most notably in estimating a notoriously difficult individual fitness [15], [16]. It also results from the availability of rich datasets collected on wild populations over several decades. Such data depth allows to study the evolution and the ecological correlates of life-history traits in the wild.

In the case of mammal species, most studies investigating the relationship between longevity and fitness have focused on females [6]–[8], [10]–[12], [17]–[19] (but see [9]). These studies, which mainly concerned large terrestrial herbivores, usually found evidence of long-lived females having a larger fitness than short lived ones (but see [7]). On the other hand, males are usually not studied as estimating their fitness is harder and often demands genetic analysis to reliably infer offspring's paternity.

The Southern Elephant Seal (*Mirounga leonina*) is the most dimorphic and polygyneous mammal among extant species. The biology of this elusive carnivore, which can spend up more than 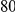
% of its lifetime at sea [20], means that seals are not observable most of the time. Moreover, when ashore during the breeding season, males fight to hold harems of numerous females. Most males never reproduce but a few mates with a large number of females [21]. Body size is a critical component for holding and fighting over a harem. Since these seals can grow all their life [22], breeding for a male depends on surviving long enough to reach an adequate size to be able to hold a harem. We may therefore expect a strong relationship between longevity and fitness in males [23]. Assessing longevity in the male Southern Elephant Seal using mark-recapture methods is extremely demanding: less than 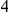
% of a cohort may survive up to 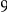 years-old [24], when they may become harem-holders [25]. Moreover the question of why theses males manage to outlive the others is left open since their at-sea behaviour remains elusive.

One way to overcome this problem is to rely on indirect methods to infer the at-sea ecology of these animals. In this respect, the study of marine mammals has greatly beneficiated from the use of stable isotopes [26]. Carbon (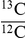) and nitrogen (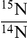) stable isotope ratio are the most commonly used elements in isotopic dietary studies. Carbon (nitrogen) stable isotopes can provide information about the diet's geographical origin (trophic position) of a consumer [27]. In the Southern Ocean, the existence of a latidudinal gradient in carbon stable isotopes across water masses [28]–[30] allows researchers to infer where elephants seals have been foraging prior to hauling out [31]. The temporal window reflected in stable isotope values depends on the sampled tissue [32]. Therefore, questions that may be addressed with stable isotopes are tied to the careful choice of an appropriate tissue.

Stable isotopic measurement of tissues that are metabolically inert after synthesis, such as teeth or baleen, can yield information on the ecology of marine mammals over their whole life [33]–[37]. Incremental tissues of teeth may further allow age estimation [38]–[40]. Measuring stable isotopes in teeth may permit to investigate the ecological correlates of longevity. In the present work, we studied the influence of foraging strategy, as inferred from carbon stable isotopes measured in tooth, on the individual lifespan of male Southern Elephant Seal breeding on îles Kerguelen, Southern Ocean. Bailleul *et al.* [31] found that males from îles Kerguelen were mainly foraging either in Subantarctic waters (mostly the Kerguelen Plateau) or on the Antarctic Plateau (see Figure S1). In a previous study [41], we investigated the ontogeny of foraging behaviour. Here we investigated whether the observed dual strategy affects the longevity of males Southern Elephant Seals.

## Results

### Growth Mixture Modelling

**Figure 1:**
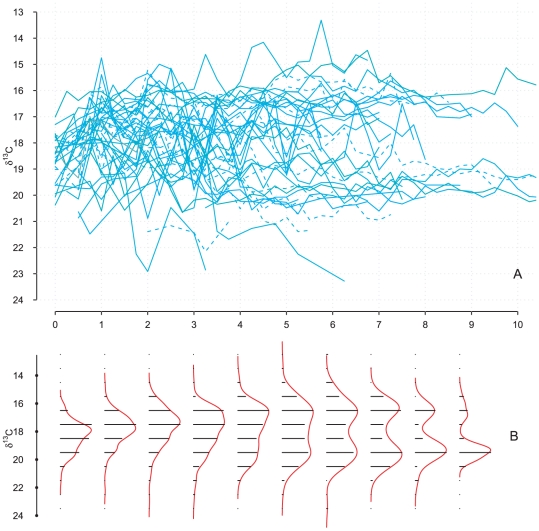
Spaghetti and density plots of the tooth 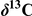 values from male Southern Elephant Seals. The distribution is unimodal up to age 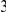 but changes to a bimodal distribution afterwards. Observations belonging to the component with the smallest mean are first in a minority but progressively increase in proportion until becoming the majority.

**Figure 2:**
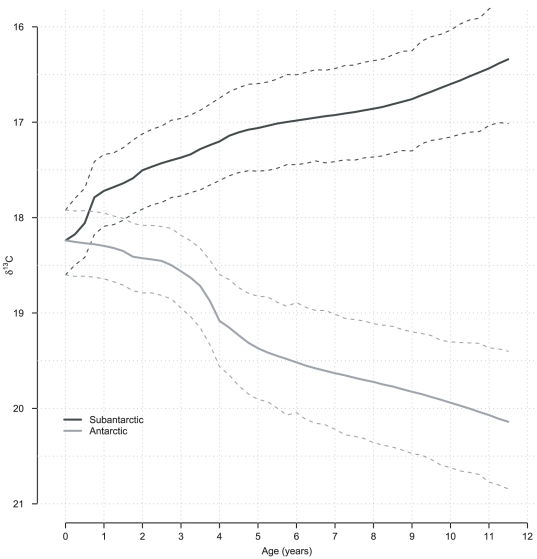
Mean 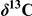 values of the two-components growth mixture in relation to Southern Elephant Seal age. Posterior means along with 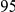
% Highest Probability Density (HPD) intervals are depicted. For advanced age classes (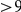 years), this model suggested an increase in mean 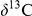 values for Subantartic foragers, and a decrease for Antarctic foragers. This effect seems artefactual in light of Figure 1 where isotopic values are stable after age 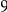. The artefact results from the restrictive assumption on the growth curve shape (see Methods).

Bailleul *et al.* [31] investigated the foraging behaviour of juveniles males using both remote-sensing tags and blood carbon isotopes. Blood 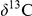 values for young small-sized males had a unimodal distribution while there was evidence of a bimodal distribution for older large-sized males. Dentin 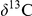 values had a unimodal distribution for individuals younger than 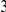 years old, and a clear bimodal distribution after that age (Figures 1 and 2). However, this approach only described the growth pattern of 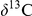 conditional on a foraging strategy. Furthermore it made the restrictive assumption that the growth curve *shape* of individuals with the same foraging strategy is identical. However this model was used for descriptive purposes, and below we present the results of an explanatory approach which aimed at identifying ecological correlates of longevity.

### Joint Modelling

**Figure 3:**
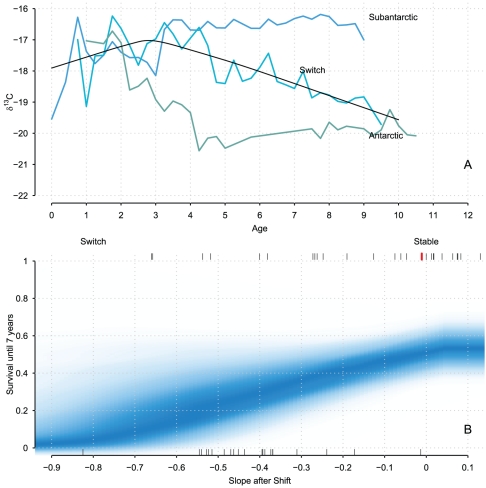
Survival curve estimated from the joint change-point/AFT model. The upper panel illustrates the different isotopic profiles observed in our data. A density strip plot of the estimated survival function is depicted on the lower panel to emphasize uncertainty [100], [101]. The probability of male Southern Elephant Seals to live up to 7 years, the mean longevity observed in our sample, is depicted as a function of the slope of the isotopic profile after an ontogenetic shift. Seals that had a stable foraging strategy were also long-lived individuals.

We adopted a joint modelling approach for analyzing lifespan [42]–[47]. We used a hierarchical change-point model [48], [49] to describe individual time-series of 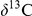 values (see Figure 3A), which enabled us to identify an ontogenetic shift between a juvenile stage and an adult one when males were committed to either an Antarctic or a Subantarctic strategy [41]. For the adult stage, the regression slope of 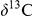 values against age is either close to zero, which means a very stable strategy of foraging in either Subantarctic or Antarctic waters, or negative reflecting the preponderance of foraging in Antarctic waters (see Figure 3A). We estimated 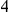 individual parameters describing a broken-stick model and subsequently used them as predictors in an Accelerated Failure Time (AFT) model [50], [51].

**Figure 4:**
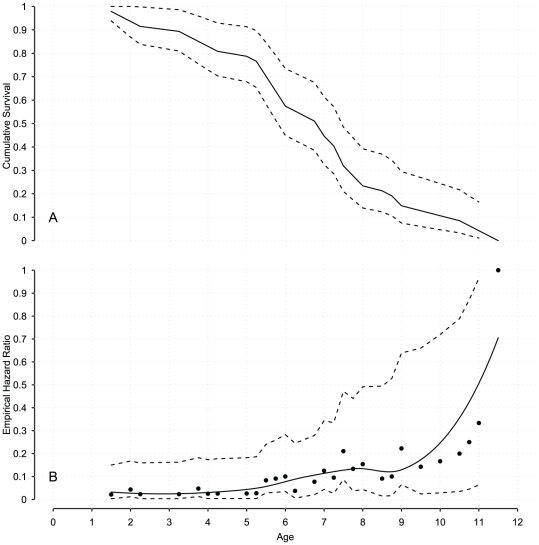
Survival analysis of male Southern Elephant Seals. The upper panel depicts the empirical Kaplan-Meier survival curves [102], with the continuous line representing the mean, and dashed lines a 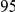
% confidence interval. The lower panel illustrates the empirical hazard ratio, along with a loess curve (continuous black line): the assumption of a monotonic hazard is reasonable as hazards are ever increasing. 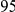
% confidence intervals (dashed lines) for the empirical hazard ratio are also represented: these intervals widen with age as the number of individuals at risk decreases in advanced age classes.

With an AFT, survival times are directly modelled, which eases the interpretations of coefficients; but a parametric distribution family must be specified [50], [51] in contrast to the semi-parametric Cox Proportional Hazard (PH) model [52]. We nevertheless opted for the AFT model and assumed survival times to follow a Weibull distribution [43], whose core assumption is a monotonic hazard function [53], which seems reasonable for these data (Figure 4B and Figure S2). We embedded the AFT within a hierarchical change-point model for 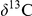 values [41] (See the annotated code in Supplementary Materials).

The best survival model was the joint change-point/survival model (Table 1), but the model fit as assessed using Kolmogorov-Smirnov test was poor (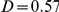, 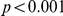, Figure S3). Further investigations also revealed modest to strong correlations (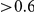) between the 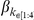 (Figure S4). Close inspection of the individual-specific parameters 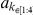 revealed that the only parameter to truly covary with longevity was 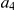, the slope after the ontogenetic shift (Figure 4B). This was further checked and confirmed with Stochastic Search Variable Selection [54] (not shown). Hence only the posterior estimate of 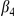 is reported in Table 2. Since 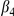 was negative, males with a very stable foraging strategy (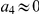) had on average a larger life-span than the other males.

**Table 1:** Accelerated Failure Time (AFT) model selection.

Model	K	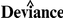	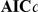	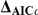	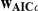
Joint	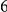	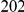 . 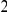	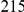 . 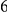	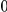 . 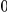	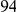 . 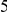
Null	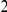	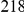 . 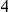	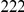 . 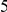	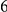 . 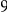	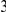 . 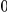
Mixture	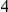	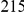 . 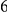	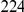 . 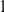	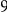 . 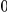	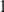 . 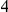
Random	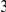	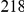 . 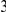	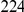 . 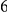	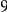 . 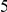	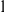 . 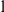

Four models were considered and we kept a ratio of number of parameters to be estimated to the number of datum close to 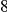. The Akaike Information Criterion corrected for small sample size (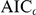) is reported. 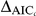 is model 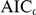 minus the minimum observed 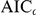, and 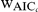 are model weights. The best model in terms of predictive ability was the joint change-point/survival model.

**Table 2:** Summary statistics for the parameters of the joint change-point/survival model.

Parameters	Median	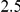 %	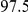 %	Interpretation
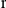	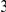 .3	_2·6_	_4·2_	Shape of the Weibull distribution
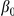	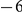 .7	_−8·6_	_−5·0_	Intercept
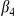	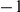 .4	_−2·8_	_−0·1_	Slope after shift

## Discussion

### Ecological correlates of longevity

As expected, male Southern Elephant Seals showed a clear mixture of two foraging strategies as they aged [31], [55]. Using carbon stable isotopic measurement from tooth, we found that some males had an Antarctic signature (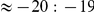
‰), while others had a Subantarctic signature (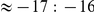
‰). The pattern in Figure 1B suggested that 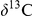 values reflective of an Antarctic signature increased in proportion over time. Such a pattern may reflect the progressive disappearance of males foraging elsewhere than in Antarctic waters, either because of an ontogenetic shift in foraging behaviour [56] or because of differential survival of males with different foraging strategies. To investigate this matter further, we adopted a joint change-point/survival modelling approach to explicitly relate the age of an individual [39] with a proxy of its foraging behaviour [34], [35].

Males foraging in Antarctic waters didn't have a longer longevity than males foraging in the Subantarctic waters. The increasing proportion of Antarctic 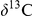 values (Figure 1) was more the result of small sample size [57] for advanced age classes and of ignoring within-individual correlation (recall that a seal of a given age can contribute up to 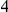 isotopic values because of the sampling design, see **Materials & Methods**). A joint modelling approach, which accounted for the longitudinal nature of our data, revealed a relationship between the stability of a foraging strategy and longevity. Seals that exhibited little variation in their tooth 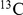 profile were also the most long-lived. A change-point model evidenced a negative correlation between the age at ontogenetic shift and the slope after this shift [41]: seals that had an early shift were constant in their foraging behaviour for the rest of their lives. Thus, this sophisticated modelling approach confirmed what an “eye-ball" analysis suggested: profiles with the smallest isotopic variation were from seals with the longest life-span (Figure 1A). The two modes that progressively appear with age on Figure 1B reflects how seals that became faithful to a foraging strategy early in life lived longer that others. Thus, the two modes in the distribution of 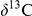 values (Figure 1B) partly arose from the selective disappearance of males with a variable 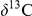 profile.

This pattern of an early shift in life associated with far-reaching consequences in later-life underscore how crucial are the first years of life in this species [58]. There was in fact a small (in magnitude) positive correlation between the (positive) slope before and the (negative) slope after the shift [41]. The positive slope before the shift was expected because weaned pups rely exclusively on maternal milk before weaning. Phociid milk is very rich in lipids [59], [60], which are depleted in the heavier carbon isotope [61]. Thus the positive slope before the shift may in part reflect the progressive independence from maternal resources [34], [40], [62], [63]. Pups which became early on independent from maternal ressources, were able to forage on their own and adopted a very stable foraging strategy. Those same pups also lived longer than the others, suggesting thereby the potential importance of early life history on latter performances [64]–[66].

Bradshaw *et al.* [67] studied the fidelity of adult female Southern Elephant Seals to their foraging grounds. Using a measure of overlap between visited zones along at least two consecutives foraging journeys, Bradshaw *et al.* [67] directly evaluated how females seemed to behave according to simple navigation rules, that is how females were “rational" in the sense that their behaviour was predictable. One major finding of this elegant analysis was the lack of a relationship between mass gain and spatial overlap between two successive foraging trips: females showed fidelity to a foraging ground irrespective of foraging success. Although Bradshaw *et al.* [67] lacked data on long-term survival and lifetime reproductive success of these females, which were of the same age-period-cohort to limit potential counfounders, they speculated that the stability shown by these females may have arisen in their early life. While our study is confined to males, it is in agreement with the results of Bradshaw *et al.* [67]. Despite a cruder spatial resolution compared to tracking data, stable isotopes enabled us to look into the ontogeny of foraging strategy in male Southern Elephant Seals, and thus to evidence how foraging fidelity was associated with longevity.

We were nonetheless surprised that the Antarctic strategy was not associated with an increased life-span. At least for females, an Antarctic strategy may yield higher fitness pay-offs. There is a latitudinal gradient in pup weaning mass with pups born in colonies closer to Antarctica having a larger weaning mass on average than pups born at lower latitude rookeries [68]. As weaning mass correlates with first-year survival [69], this suggests that resources in Antarctic waters may be more profitable. Chaigne *et al.* [70] analysed blood stable isotopes of juvenile Southern Elephant Seal males. Their study design was cross-sectional, but Chaigne *et al.* [70] showed that older males, as assessed from their body length, were more likely to forage in Antarctic waters compared to younger ones. They have interpreted this pattern as an ontogenetic shift in foraging grounds, which is consistent with the stable isotope analysis of dentin [41]. Foraging in Antarctic waters thus seems to be favored by bigger males, possibly because of higher fitness pay-offs.

Yet our longevity data did not suggested an increased survival of Antarctic foragers. Unlike females which remained in the marginal sea-ice zone, juvenile Southern Elephant Seal males from îles Kerguelen readily foraged in the pack ice [55]. Getting trapped in the ice is a potential cause of mortality that males foraging over the Kerguelen Plateau or at the Polar Front do not face [31]. Our present analysis does not point to different foraging grounds influencing male life-span. The pattern uncovered is one of the benefit of a very stable foraging strategy with no deviation from an early age in life. This pattern may lend support to a spatial familiarity hypothesis [71], [72], although a direct experimental test of such an hypothesis is currently not possible with Southern Elephant Seals.

That isotopic profiles covaried with longevity thus suggests that variability in foraging strategy is costly. Such costs may arise from unfamiliarity with novels environments, such as a greater susceptibility to predators, or increased travel costs. A non-exclusive alternative is that some individuals were more able to extract resources efficiently from the environment, either in Antarctic or Subantarctic waters. None of these interpretations suppose a strategy to be superior to the other in terms of fitness return. However, the second interpretation implies that seals which are less efficient to acquire resources may switch between foraging strategies while those which are efficient have no reason to do so. Under this latter interpretation, the 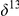
C profile of seals may reflect their ‘quality’, quality being understood as a static trait that positively correlated with fitness [73]. We chose the term quality over fitness here as the reproductive success of males in our sample is unknown. Yet given the breeding biology of male Southern Elephant Seals, living long enough to grow large and defend a harem is a pre-requisite to contribute offspring to the next generation [23].

### Limitations

A potential confounding factor in our data is that teeth were sampled from dead animal on beaches, thus we had to assume our sample was representative of the larger population of all males on îles Kerguelen. Assuming otherwise would imply that male found dead on beaches were different than those dying at sea. The average longevity in our sample was 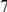 years, and only 5 males were older than 10 years (Figure S5). In their study on reproduction costs on Sea Lion Island (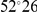
′S, 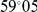
′W), Galimberti *et al.* [25] found only 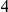 males out of 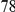 (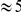
%) to be older than 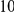 years old, while McCann [74] reported a proportion of 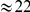
% for South Georgia (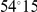
′S, 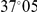
′W). The observed proportion in our sample was 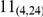
%, compatible with both the Sea Lion Island and South Georgia estimates. Age in our study was estimated from teeth growth layers: there is an uncertainty associated with age (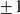 year). It is, however, very small [40] and cannot reverse the observed pattern. Defining a species' longevity as the time by which 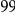
% of a cohort has died [75], the specific longevity of male Southern Elephant Seals is 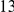 years [76]. The oldest male in our sample was estimated to be 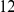 years old, which suggested that our sample did not seem atypical with respect to old age classes.

Of concern may be the lack of fit of the AFT model to the data. Even our best model in term of 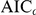 did not provide an adequate fit to the data (see Figure S3). Yet it has been argued that the poor *predictive* ability is an intrinsic feature of survival models with realistic parameter values [77]. Our approach here was explanatory rather than predictive [78]. The joint model clearly captured some aspect of the data unaccounted for by the Null model given its large Akaike weight. Further model checking revealed that this model was overparametrized, but still performed better than the null model despite the penalty for the larger number of (unnecessary) parameters. The mixture model also has a larger likelihood than the null model, but its larger number of parameters put it on a par with the null model (similar Akaike weights). Thus the data suggest an effect of the foraging strategy that we seemed to have picked up best with a change-point model.

### Conclusion

Stable isotopes, while lacking the fine scale resolution of tracking data, can reveal surprising ecological features of a species [26]. However, this crudeness may proved a strength: by summarizing a whole foraging trip with a single number, isotopic data provided an integrative measure that can be easily fed into a model specifically tailored to the problem at hand. Using an appropriate tissue, stable isotopes can also provide longitudinal data [33]–[37].

The explicitly modelling of foraging strategy ontogeny in male Southern Elephant Seal via a change-point (or broken-stick) model of 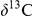 values revealed how long-lived animals were those faithfull to a foraging strategy from an early age. This finding emphasizes the importance of early life in life-history trajectories. It also suggests that variability in foraging strategies might be costly for adult male Southern Elephant Seals from îles Kerguelen. In other words, faithfulness to a foraging strategy predicted a long-life for males.

Studying of the life-span of wild animals is a difficult endeavour: ecological correlates can be uncovered but a large amount of variation usually remains unaccounted for in the analysis [79]. This is unsurprising in light of all the potential factors, related to fitness or accidental, that may affect an individual throughout its whole life [3], [12], [80].

## Materials and Methods

### Ethics Statement

The ethics committee of the French Polar Institute (*Institut Paul Emile Victor* - IPEV) approved this study. All animals in this study were cared for in accordance with its guidelines. This study is part of a national research program (no. 109, H. Weimerskirch and the observatory *Mammifères Explorateurs du Milieu Océanique*, MEMO SOERE CTD 02) supported by the French Polar Institute (IPEV). The approval ID for this study is IPEV research program no. 109, which is evaluated every year by the ethics committee of the French Polar Institute.

### Sample Collection

Teeth were collected from male elephant seals that died of natural causes on îles Kerguelen (49 30′S, 69 30′E), Southern Indian Ocean. Canines grow continuously throughout the whole life of males without closing of the pulp cavity, allowing for age determination [81]. Canines from 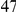 males were analyzed and sampled for isotopic analysis (see [40] for a full description of age determination and isotopic sampling). Briefly, each tooth was cut longitudinally and observed under diffused light to count growth layers. The alterning pattern of two opaque and two translucent growth layers corresponds to the annual biological cycle of Southern Elephant Seals [39]. Translucent bands are enriched in vitamin D and synthesized when seals are ashore to breed and to molt, while opaque ones are synthesized when at sea [82]. Within a year, a Southern Elephant Seal comes onshore to breed, returns to the sea, then comes onshore to moult and forages once more at sea before the next breeding season. Thus each growth layer was assumed to correspond to one forth of a year [40]. Each growth layer was sampled for 1 mg of bulk dentin using a 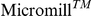 sampler (ISEM, Université de Montpellier 2).

As a recent study raised concerns about non-linear offsets of organic %C, %N and 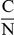 after acid treatment [83], we forwent any acid (or demineralization) treatment prior to isotopic measurement. As a result, the measured 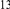
C is a mixture of organic carbon with a small amount of inorganic carbon. To test the impact of the inorganic fraction, Martin *et al.* [40] compared acid-treated and untreated samples but found no differences (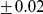). Schulting *et al.* [84] found similar 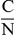 ratios between bulk dentin and collagen, with a lower carbon and nitrogen contents in bulk dentin most likely due to the mineral fraction. Here we assumed that the impact of the mineral fraction is negligible.

For measurement of carbon stable isotopes and 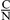 ratio, a total of 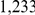 dentin increments over the 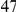 male teeth were analyzed. Elemental C and N contents (%) and carbon isotope values were measured by dry combustion using a Euro Vector 3000 Elemental Analyzer coupled with a Micromass Optima Isotope Ratio Mass Spectrometre (ISEM, Université de Montpellier 2). Results are expressed in percentage of powder weight (Total C and N) and as 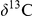 (‰) with respect to the Vienna-Pee Dee Belemnite standard using the conventional delta notation:
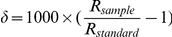
where 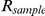 and 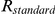 refer to the 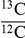 ratios of sample and standard, respectively. Analytical precision was better than 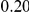
‰. We used 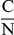 ratio thresholds of bone and tooth collagen (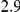 to 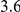) as criteria for the identification of diagenetic alteration [85]; assuming that total dentin, whose organic phase is mainly collagen and water [86], has the same 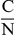 ratio than bone and tooth collagen. From the 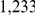 analyzed sampled, 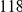 were discarded, yielding a final sample size of 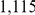 isotopic values from 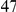 males. Given the alterning pattern of tooth growth layers, up to 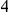 isotopic measurements were available for a given year of life.

### Growth Mixture Modelling

Data were first analyzed using growth mixture models [87]. This approach respects the longitudinal nature of our data, and is superior to simple mixture models. The aim of this modelling exercise was primarily descriptive, that is we aimed at summarizing our data. Bailleul *et al.* [31] found that males from îles Kerguelen were mainly foraging either in Subantarctic waters (mostly the Kerguelen Plateau) or on the Antarctic Plateau (see Figure S1). We thus assumed a two-components mixture. For the 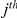 male, we modelled its blood isotopic signature at age 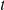 as:
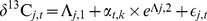
(1)where 
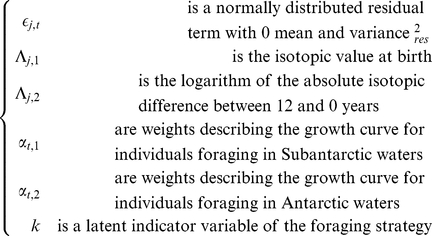



For model identification purposes, we constrained 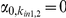, 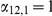 and 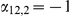. In addition, to circumvent any label switching issues, we further constrained the isotopic value of Antartic foragers to be lower than that of Subantartic foragers. Practically, we allowed the batch of weight coefficients of the first component of the mixture to be positive (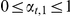) but forced the second batch to be negative (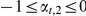). Finally, we assumed the growth curves to be isotonic: for all age 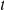, 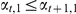 and 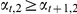. Isotonicity translates an assumption about isotopic equilibration to a foraging habitat signature. A crucial assumption of this approach is that the growth curve *shape* of individuals with the same foraging strategy is identical, which is not reasonable given the variety of profiles observed in Figure 1. To relax this assumption, we used a hierarchical random change-point model [41].

### Joint Modelling

Change-point models aim at finding an abrupt rupture in a time-series. The time-series is assumed to be the juxtaposition of piece-wise linear homogeneous segments, each segment separated from the next by a change-point. These models are very flexible as they allow specifying different probability distributions to describe different parts of a time series. Different curve shapes can thus be generated. Change-point models thus seem appropriate to describe ontogenetic shifts [41], [56]. A time-series is summarized in 4 parameters: a value at the change-point, the timing of the change-point, and a slope before and after the change-point. In a previous paper [41], we used a hierarchical change-point model to describe individual time-series of 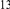
C measurement in Southern Elephant Seal teeth and found evidence of ontogenetic shifts. Here, we assessed the impact of these ontogenetic shifts on longevity.

Because teeth were sampled from dead animals that were subsequently aged, all survival times are observed: there is no censoring in the data. Denoting 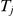 the survival time of the 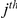 male, we assumed the 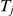 to follow a Weibull distribution of parameters 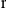 and 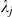:
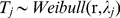
(2)

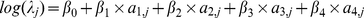
(3)where 
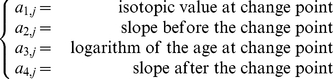


The shape parameter 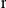 controls the hazard rate with 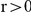 (resp. 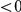) describing an increasing (resp. decreasing) hazard with time. With our data, we expected 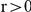 (Figure 4). The parameters 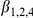 then quantify the association between foraging location (via 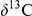) and longevity. The parameter 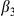 captures the relation between age at ontogenetic shift and longevity. The parameter we are particularly interested in is 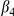 as it reflects the correlation between the stable foraging habitat of adults and their longevity. In the AFT, a positive 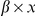, where 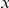 is the covariate value, accelerates the occurrence of the event (death), while a negative value retards it. A negative 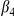 means that individuals foraging in two different water masses (negative slope) die earlier than those males which have a very stable strategy (null slope). The joint modelling approach is especially suited here as it makes use of all the available data and the Bayesian framework guarantees that uncertainties in estimating the 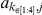 are taken into account [43], [46].

To assess the appropriatedness of our joint model, we compared it with 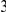 other AFT survival models:

- a null model with with no individual-level covariate;
- a random-effect model wherein an individual-specific deviation from the mean 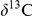 value was incorporated as a covariate for the AFT model; and lastly
- a mixture model wherein each male was first assigned to a group depending on its mean 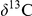 value and then group membership was included as a covariate for the AFT model.

Strictly speaking, both the random and mixture models are also joint models, as time-series of 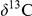 values were used to derived predictors for the survival analysis. Model comparison were done using the Akaike Information Criterion with a small sample correction, 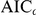 [88]. Our sample size is modest (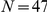) and the most complex AFT model considered had 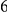 parameters, keeping the ratio of sample size to parameter number above 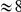 which is slightly below the recommended 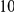 [53]. The goodness-of-fit of the selected model was checked by comparing the predicted longevity with the observed one using Kolmogorov-Smirnov test. Finally, we investigated in a preliminary analysis whether males born before and after the 1970s population crash [89] had different longevity and found none (Likelihood Ratio Test: 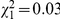, 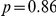).

Growth layer synthesized while seals were ashore were kept in all analyses. These layers may differ from the others since Southern Elephant Seals fast on land. Retaining these layers may add measurement error linked to physiological processes. We then compared for each seal the distribution of isotopic values measured in dentin synthesized ashore *versus* at-sea with a Kolmogorov-Smirnov test. Except for one individual, there was no statistically significant differences (See Table S1). Excluding this individual did not change our results. Balasse *et al.* [90] estimated the isotopic equilibration of dentin after diet change to take between 1 to 4 months in cattle (*Bos taurus*). This time-span is commensurate with, if not longer than, the typical haul-out duration of a Southern Elephant Seal. Assuming similar equilibration time for cattle and elephant seals, this may explain why no statistically significant differences were found (See Table S1).

### Software

We used *winBUGS* [91] called from *R* [92] with the package *R2WinBUGS* [93]. Weakly informative priors were used [94], [95]. For the growth mixture model, we used a uniform prior for the residual variance; Normal priors for regression parameters on the natural scale; the default Student-*t* prior of [95] for regression parameters on a logarithmic scale; and a beta(2,2) prior for the mixing proportion. We used the SVD prior of Tokuda *et al.* [96] for the covariance matrix controlling the 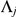: random orthogonal matrices were generated as described in Anderson *et al.* [97]. Three chains were initialized with overdispersed starting values. After appropriate burn-in (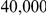 iterations) and thinning of the chains (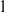 value every 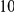 iterations stored), convergence was assessed using the Gelman-Rubin convergence diagnostic [98] with the *coda* package [99]. For the joint model, Authier *et al.* [41] detailed the hierarchical change-point model fitted to the isotopic data. We used for the AFT model the default Student-*t* prior of [95] for the parameter 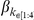, and a uniform prior bounded between 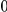 and 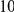 for 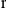. Three chains were initialized with overdispersed starting values. After appropriate burn-in (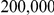 iterations) and thinning of the chains (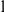 value every 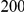 iterations stored), convergence was assessed using the Gelman-Rubin convergence diagnostic [98] with the *coda* package [99]. Unless stated otherwise, posterior mean and standard error of the mean are reported, either with its standard error (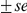) or with 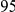
% Highest Probability Density (HPD) intervals (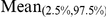). Inferences are based on a posterior sample of 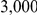 iterations. Annotated *BUGS* code is available in Text S1 (Growth Mixture Models) and Text S2 (Joint Change-point/Survival Model).

## Supporting Information

Figure S1 **Satellite tracking of Southern Elephant Seal males breeding on îles Kerguelen.** Examples of 24 tracks are represented (solid blue lines) to illustrate the different strategies: males mainly forage in the Antarctic Zone, on the Kerguelen Plateau or in Subantarctic waters (waters lying between the Sub-Antarctic Front and the Southern Antarctic Circum-Polar Front). Îles Kerguelen (Ker), and the Antarctic coastline's contour are depicted in black, and while the 2000 metres isobath is depicted in light grey. Dotted lines symbolized fronts [103], within the Southern Ocean: SubTropical Front (STF), Sub-Antarctic Front (SAF), Polar Front (PF) and Southern Antarctic Circum-Polar Front (SACCF). Marginal histograms of localisations are represented on the side to illustrate the different strategies.
(EPS)

Figure S2
**Checking the appropriatedness of the Accelerated Failure Time model.** With the BUGS parametrization:
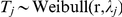


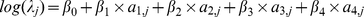
The hazard function is: 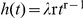, and the survival function is: 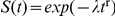. An empirical test for the Weibull distribution is provided by the plot of the estimate of 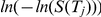 *versus* 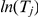, which should give a straight line: 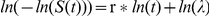.(EPS)

Figure S3
**Goodness-of-fit of the joint change-point/survival model.** The selected model, although being the best in term of predictive performance among the set of competiting model, could be still improved. A Kolmogorov-Smirnoff goodness-of-fit test indicated that the considered covariates were not a sufficient set for these data (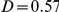, 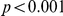).(EPS)

Figure S4
**Correlations between the predictors of the Accelerated Failure Time survival model.** The large correlations means that these parameters are not independent. Further model checking revealed that only 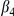 covaried with longevity.(EPS)

Figure S5 **Histogramm of the observed longevity of males included in the sample.**
(EPS)

Table S1 **Kolmogorov-Smirnoff tests for comparing dentin layers grown onshore or at sea.** For only one individual was there a significant difference. Removing this individual did not change the analysis.
(XLS)

Text S1 ***WinBUGS*** **code to fit the Growth Mixture Model.**
(TXT)

Text S2 ***WinBUGS*** **code to fit the joint Accelerated Failure Time\Change-point Model.**
(TXT)
